# Assessment of medical students’ clinical performance using high-fidelity simulation: comparison of peer and instructor assessment

**DOI:** 10.1186/s12909-021-02952-w

**Published:** 2021-09-25

**Authors:** Ji Hye Yu, Mi Jin Lee, Soon Sun Kim, Min Jae Yang, Hyo Jung Cho, Choong Kyun Noh, Gil Ho Lee, Su Kyung Lee, Mi Ryoung Song, Jang Hoon Lee, Miran Kim, Yun Jung Jung

**Affiliations:** 1 Office of Medical Education, Ajou University School of Medicine, Suwon, South Korea; 2 Department of Medical Humanities and Social medicine, Ajou University School of Medicine, Suwon, South Korea; 3 Department of Gastroenterology, Ajou University School of Medicine, Suwon, South Korea; 4 Ajou Center for Clinical Excellence, Ajou University School of Medicine, Suwon, South Korea; 5 Department of Pediatrics, Ajou University School of Medicine, Suwon, South Korea; 6 Department of Obstetrics & Gynecology, Ajou University School of Medicine, Suwon, South Korea; 7 Department of Pulmonary and Critical Care Medicine, Ajou University School of Medicine, Suwon, South Korea

**Keywords:** peer assessment, clinical performance, high-fidelity simulation, medical student

## Abstract

**Background:**

High-fidelity simulators are highly useful in assessing clinical competency; they enable reliable and valid evaluation. Recently, the importance of peer assessment has been highlighted in healthcare education, and studies using peer assessment in healthcare, such as medicine, nursing, dentistry, and pharmacy, have examined the value of peer assessment. This study aimed to analyze inter-rater reliability between peers and instructors and examine differences in scores between peers and instructors in the assessment of high-fidelity-simulation-based clinical performance by medical students.

**Methods:**

This study analyzed the results of two clinical performance assessments of 34 groups of fifth-year students at Ajou University School of Medicine in 2020. This study utilized a modified Queen’s Simulation Assessment Tool to measure four categories: primary assessment, diagnostic actions, therapeutic actions, and communication. In order to estimate inter-rater reliability, this study calculated the intraclass correlation coefficient and used the Bland and Altman method to analyze agreement between raters. A t-test was conducted to analyze the differences in evaluation scores between colleagues and faculty members. Group differences in assessment scores between peers and instructors were analyzed using the independent t-test.

**Results:**

Overall inter-rater reliability of clinical performance assessments was high. In addition, there were no significant differences in overall assessment scores between peers and instructors in the areas of primary assessment, diagnostic actions, therapeutic actions, and communication.

**Conclusions:**

The results indicated that peer assessment can be used as a reliable assessment method compared to instructor assessment when evaluating clinical competency using high-fidelity simulators. Efforts should be made to enable medical students to actively participate in the evaluation process as fellow assessors in high-fidelity-simulation-based assessment of clinical performance in situations similar to real clinical settings.

**Compliance with Ethical Standards**.

This study was approved by the Institutional Review Board (IRB) of Ajou University Hospital (Ethics Consent No. AJIRB-SBR-SUR-20-255).

## Disclosure

On behalf of all authors, the corresponding author states that there is no conflicting interest.

## Background

The evaluation of clinical performance in medical education is shifting to competency-based assessment [1]. Simulation has been used in clinical education and evaluation of clinical competency [2, 3]. Simulation-based assessment (SBA) enables direct assessment of clinical performance and provides an opportunity to simultaneously assess knowledge, clinical reasoning, and teamwork [4]. SBA has the advantage of being able to present scenarios that are not visible and allow students to actually perform the entire treatment process in a safe environment with no risk to patients, which provides treatment evaluation and feedback [5, 6]. In particular, high-fidelity simulators are a reliable and valid method for evaluating clinical performance [2].

Recently, the importance of peer evaluation has been highlighted in healthcare education, with the value of peer assessment examined in various healthcare fields, such as medicine, nursing, dentistry, and pharmacy [7]. Peer assessment refers to evaluating and providing feedback on the performance of people of similar levels of competence [8]. Assessing a peer’s performance as an evaluator not only reflects on one’s own performance but also aids in clinical skill learning [9]. In addition, participating in the peer assessment process provides a more objective perspective based on evaluation criteria, ultimately improving clinical performance [10]. However, there are concerns that proper assessment of peers’ competence may be challenging due to personal or competitive peer relationships [11]. Therefore, the reliability and validity of peer assessment compared to instructor assessment in medical education requires continuous reevaluation. And the importance of training to develop appropriate assessment skills should be considered in order to utilize peer assessment [12].

Most prior studies comparing and analyzing peer and instructor assessment of clinical performance focused on performance in the objective structured clinical examination (OSCE) [7]. But, there is scarce research analyzing performance of high-fidelity-simulation-based assessment, which has been used recently to improve patient safety and quality of care. Therefore, this study aimed to investigate the reliability of peer assessment of clinical performance in the field of medical education compared to instructor assessment. This study posed the following research questions:

1. What is the reliability of scores between peer and instructor assessment of clinical performance using high-fidelity simulation?
2. What is the degree of agreement rate between peer and instructor assessment scores of clinical performance using high-fidelity simulation?

## Methods

### Design

This was a quantitative study to verify the reliability of peer assessment as an evaluation tool for clinical performance based on simulation and compare assessment scores between peers and instructors.

### Ethical considerations

This study was approved by the Institutional Review Board (IRB) of Ajou University Hospital (Ethics consent No. AJIRB-SBR-SUR-20-255).

### Setting

This study was conducted at Ajou University School of Medicine in Korea during clinical practice in pulmonary and gastroenterology in 2020. The clerkship in pulmonary and gastroenterology medicine was conducted for one week each. And the assessment was performed once on the last day of the clinical practice of each department, and a total of two assessments were made.

Simulation practice was conducted using a high-fidelity simulator (Laerdal’s SimMan 3G patient simulator). It was carried out in the simulation lab equipped with a wireless touch screen monitor device that can provide X-rays, media, and lab results according to the programmed scenario connected to the simulator, and a storage box including the equipment necessary for performing the technique. The instructor monitored and evaluated the students’ performance in the monitoring room with one-side mirror connected to the simulation lab. The peer assessment was conducted in a way that students evaluate the performance of their fellow students by transmitting the status of the entire practice process wirelessly in real time through a dedicated audio/video system integrated with SimMan 3G software.

Each department contained 17 groups consisting of two to three students into a group, that is, a total of 34 groups were evaluated. Assessment was based on the performance of the entire group, not individual assessments. Group assignment and order of simulation were randomized. Each group was randomly assigned one scenario out of six pulmonary and seven gastrointestinal disease scenarios. Students experienced one case each for pulmonary and gastrointestinal diseases. Each scenario consisted of a case focused on clinical presentation. Pulmonary disease scenarios included symptoms of acute respiratory distress and consisted of pneumonia with parapneumonic effusion, chronic obstructive pulmonary disease with acute exacerbation, pulmonary thromboembolism, heart failure with pulmonary edema, spontaneous pneumothorax, and iatrogenic tension pneumothorax. Gastrointestinal scenarios included melena, jaundice, hematemesis, diarrhea, abdominal pain, abdominal distension, and abdominal mass.

The process consisted of a pre-briefing, simulation, and debriefing. During pre-briefing, students prepared the equipment and checked the patient’s main symptoms and data on current medical history. Simulation proceeded for 10 to 15 min, followed by a debriefing and reflections of the entire process. The assessment of clinical performance was conducted based on performance in the simulation. Two or three students performed clinical performance on one simulator patient, and evaluation was also conducted as a group rather than an individual. Students conducted peer-assessment on the performance of one group (not their own), and two professors evaluated the performance of all groups.

### Participants

This study used the assessment data of 37 students (26 males and 11 females) excluding three who lost their response data in some questions among 40 fifth-year medical students and two professors in charge of simulation of pulmonary and gastroenterology medicine at Ajou University School of Medicine in 2020.

### Instrument

This study used a modified version of the Queen’s Simulation Assessment Tool (QSAT) developed by Hall et al. to assess medical students’ clinical performance [13]. QSAT measures the clinical competency of postgraduate emergency medicine trainees. It was used in this study as it was judged that it could be used to measure the performance of medical students in clinical practice. This study evaluated four categories: primary assessment, diagnostic actions, therapeutic actions, and communication. The peer evaluation checklist is shown in Table 1. The instructor’s checklist included more detailed items (see the pneumothorax evaluation table for pulmonary cases presented in Table 2 for an example). This assessment tool used a five-point Likert scale measured from 1 (inferior) to 5 (superior). Instructor assessment scores were calculated using the average score of the items in each category in order to analyze peer and instructor assessments using the same format.

**Table 1:** Clinical performance checklist (peer assessment form)

Domain	Item
Primary assessment	Did the students obtain an accurate medical history?
Diagnostic actions	Were physical examinations, examination prescriptions, and result reading properly conducted?
Therapeutic actions	Has an appropriate treatment plan been established and prescribed for the patient?
Communication	Was therapeutic communication with colleagues, patients, and caregivers conducted?

**Table 2:** Clinical performance checklist (sample instructor assessment form: Pneumothorax)

Domain	Item
Primary assessment	Washing hands, checking patient name
	Checking chief complaint
	Measuring and identifying vital signs, connecting a monitor with an ECG
Diagnostic actions	Checking the medical history related to chief complaint
	Performing a lung examination (inspection, palpation, percussion, auscultation)
	Blood test (ABGA, cardiac enzyme, pro-BNP, CBC, ESR, CRP, procalcitonin) prescription, patient’s consciousness evaluation, chest X-ray prescription, ECG prescription, reassessment of dyspnea, reassessment of vital signs
Therapeutic actions	Planning for the treatment of dyspnea (admission, oxygen therapy prescription, checking the results of an examination, increasing oxygen administration, chest tube insertion, intravenous prescription)
Communication	Active listening to the patient
	Encourage ideas and opinions from colleagues
	Description of patient condition, required examinations, and results

### Data analysis

In this study, data analysis was conducted using jamovi 1.2.25 version program. In order to assess reliability between raters, the intraclass correlation coefficient (ICC) was calculated, and the Bland and Altman method was used to estimate the degree of agreement rate between raters [14]. The Bland–Altman plot is a graph to visually examine differences between values obtained by two measurement methods or between the estimated and actual values [14]. It presents a distribution of scatterplots of the mean differences between assessors, indicating a 95 % limit of agreement (LOA). LOA is used to estimate natural variations between assessors, which can be reliable with narrower widths [15]. In addition, if the measured value is outside the 95 % confidence interval rather than around the line of 0, it indicates that the mean is a systematic error.

An independent t-test was conducted to analyze differences in evaluation results between peer and instructor assessment.

## Results

The results of the reliability analysis between assessors are presented in Table 3. The intra-class correlation coefficient (ICC) value was 0.675 (CI 0.349 ~ 0.838) in ICC1 type (one-way random effects), 0.685 (CI 0.439 ~ 0.823) in ICC2 type (two-way random effects), and ICC3 type (two-way mixed) showed a level of 0.685 (CI 0.436 ~ 0.824), which was statistically significant as *p* < .001. As a result of checking the ICC2 and ICC3 values to obtain the reliability values of the two evaluators, the ICC value was found to be 0.6 or higher. ICC values of 0.6 or higher can be interpreted as a significantly high consistency level among assessors [16]. The agreement between the two groups of raters was 68.5 %, indicating that reliability between raters was high.

**Table 3:** Inter-rater reliability between peer and instructor assessment

model	measures	type	ICC (95 % CI)
One-way random	Agreement	ICC1	0.675(0.349 ~ 0.838)
Two-way random	Agreement	ICC2	0.685(0.439 ~ 0.823)
Two-way mixed	Consistency	ICC3	0.685(0.436 ~ 0.824)

**Fig. 1:**
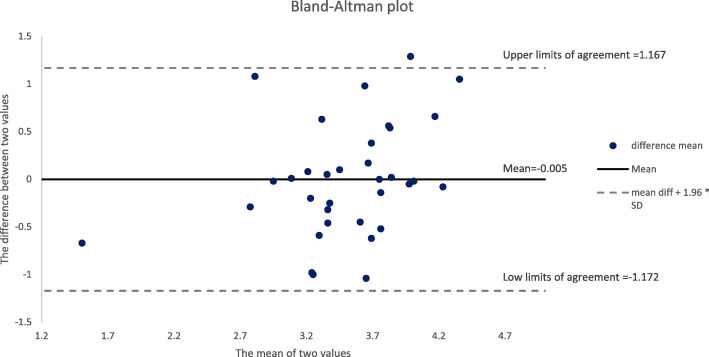
Differences in checklist scores given by peer and instructor plotted against the mean.

The results of the Bland–Altman plot for differences in means between the two groups of raters are presented in Table 4; Fig. 1.

**Table 4:** Limits of agreement between peer and instructor assessment

		95 % Confidence Interval	
	**Estimate**	**Lower**	**Upper**
Bias (n = 34) (M=-0.0048, SD = 0.59621)	-0.00475	-0.213	0.203
Lower limit of agreement [-0.0048-(0.59621*1.96)]	-1.17332	-1.532	-0.814
Upper limit of agreement [(0.59621*1.96)-0.0048]	1.16381	0.805	1.523

The results indicated that the measured values were distributed within the range of -1.17 to + 1.16 of the limit of agreement (dotted line) and gathered around the mean. Therefore, the degree of agreement between the raters was high and reliable.

The differences in assessment scores between peers and instructors are shown in Table 5.

**Table 5:** Differences in clinical performance scores between peers and instructors

		Mean ± SD		t	*p*
Primary Assessment	Peer	3.71 ± 0.69	-0.593		0.555
	Instructor	3.80 ± 0.58			
Diagnostic Actions	Peer	3.37 ± 0.80	-1.319		0.192
	Instructor	3.61 ± 0.66			
Therapeutic Actions	Peer	3.24 ± 0.97	0.940		0.351
	Instructor	3.03 ± 0.79			
Communication	Peer	3.66 ± 0.71	0.633		0.529
	Instructor	3.56 ± 0.66			
Total	Peer	3.50 ± 0.68	-0.033		0.974
	Instructor	3.50 ± 0.51			

The peer rating score was 3.50 ± 0.68, while the instructor evaluation score was 3.50 ± 0.51, indicating no statistically significant differences. Likewise, there were no significant differences between peer and instructor assessment for the categories of primary assessment (peer vs. instructor: 3.71 ± 0.69 vs. 3.80 ± 0.58), diagnostic actions (peer vs. instructor: 3.37 ± 0.80 vs. 3.61 ± 0.66), therapeutic actions (peer vs. instructor: 3.24 ± 0.97 vs. 3.03 ± 0.79), and communication (peer vs. instructor: 3.66 ± 0.71 vs. 3.56 ± 0.66).

## Discussion

The results of this study showed that peer and instructor assessment had a significant degree of agreement in evaluating the clinical performance of medical students. The accuracy and reliability of peer assessment suggests that peer assessment can be a valuable source. The high degree of agreement among evaluators found in this study, which showed strong correlation between peer and instructor assessment scores on history taking, physical examination, and diagnostic skills of medical students. This finding suggests that peer and instructor evaluations of students’ clinical performance show a tendency to be consistent.

To the best of our knowledge, this is the first research to compare peer and instructor assessment using high-fidelity simulation for medical students. Most of the previous studies with comparative analysis of evaluation scores between peer and instructor assessment for medical students were using OSCE. Previous studies showed mixed results. Several studies comparing peer and instructor assessment scores using the OSCE for medical students showed higher peer assessment scores than instructor assessment scores [17–19]. Likewise, a study that analyzed differences in peer and instructor assessment for history-taking communication skill of medical students showed that peer assessment was more lenient [20]. And another study reported that peer evaluation was a more appropriate evaluation for evaluating interpersonal skills than clinical skills [10]. On the contrary, other studies showed strong correlation between peer and instructor assessment scores in medical students [21, 22]. The present study is in line with the latter findings. The different results depending on the research show that it is necessary to continuously verify whether peer assessment is as reliable and valid assessment method as instructor assessment in medical education. This study confirms that peer assessment is a reliable method for evaluation of clinical skills, such as diagnosis and treatment planning, as well as interpersonal skills, such as communication.

Students in the clinical practice process may have areas where they lack skills in clinical performance or evaluation. Peer assessment is a useful learning activity that allows one to review one’s own knowledge and clinical skills through observing and evaluating the performance of others [9]. In other words, it is meaningful as a learning tool in that students learn through the process of participating in evaluation as assessors [8]. Therefore, it can be inferred that using high-fidelity simulation for assessment provides students the experience of observing the performance of their peers in a clinical setting similar to the real one, which has an educational effect.

This study had a limitation. The assessment checklists of peers and instructors were not identical. The detailed items of each evaluation area were not disclosed in the assessment table, as students were both assessors and assessees. A more detailed and concrete evaluation checklist should be created and analyzed in order to more accurately determine the reliability of student versus instructor assessment. In the evaluation of clinical performance, providing feedback is considered as important as providing a quantified score [23]. Follow-up studies confirming the value of peer assessment should consider feedback in order to further enhance the usability of peer assessment as an evaluation tool. The design of this study is to evaluate the performance of clinical performance in a simulation setting in groups of 2–3 students. Therefore, it has limitations in that it was not possible to evaluate the clinical performance of individual students. Also, there is a limitation in that the influence of the instructor’s rating was not considered. If multiple groups are evaluated, the effect may be greater, so it will be necessary to design the study to control the influence of the instructor variable in follow-up studies.

## Conclusions

This study examined to confirm the reliability between peer and instructor assessment in the evaluation of medical student’s ability to perform medical treatment using high-fidelity simulation. This study revealed that peer assessment can be used as a reliable assessment method compared to instructor assessment. In addition, this study found no significant differences between peer and instructor assessment of students’ clinical performance in areas of primary assessment, diagnostic actions, therapeutic actions, and communication. This study indicates that peer assessment is a reliable method for evaluating clinical competency using a high-fidelity simulator. Efforts are required to actively engage medical students in clinical competency evaluation process using high-fidelity simulation, which can assess performance in a setting similar to real clinical settings.

## Data Availability

The dataset used during the current study is available from the corresponding author upon reasonable request.

## Abbreviations

SBA: Simulation-based assessment
OSCE: Objective Structured Clinical Examination
QSAT: Queen’s Simulation Assessment Tool
ICC: Intraclass correlation coefficient
LOA: Limit of Agreement
